# A Microbial Fermentation Mixture Primes for Resistance Against Powdery Mildew in Wheat

**DOI:** 10.3389/fpls.2019.01241

**Published:** 2019-10-09

**Authors:** Tony Twamley, Mark Gaffney, Angela Feechan

**Affiliations:** ^1^School of Agriculture and Food Science, University College Dublin, Dublin, Ireland; ^2^Alltech Crop Science, Alltech European Bioscience Centre, Dunboyne, Ireland

**Keywords:** priming, powdery mildew, resistance, wheat, fermentation, defence gene

## Abstract

Since many fungal pathogens develop resistance to fungicides, novel and low-cost alternative methods to improve plant health and fitness need to be developed. An approach to improve productivity in crops is to stimulate the plant’s own defence mechanisms *via* priming. Therefore, we investigated if a fermentation-based elicitor could prime plant defences against powdery mildew in wheat by inducing the expression of endogenous defence-related genes. Wheat seedlings were spray-treated with a fermentation-based elicitor 8 days prior to inoculation with powdery mildew. Disease assays showed a significantly reduced number of powdery mildew pustules were formed on wheat treated with the mixed elicitor. *In vitro* sensitivity assays tested the ability of powdery mildew conidia to germinate on agar amended with the fermentation-based product and concluded that fungal germination and differentiation were also inhibited. Tissue samples were taken at time points pertaining to different developmental stages of powdery mildew infection. Significantly higher expression of *PR* genes (*PR1*, *PR4*, *PR5*, and *PR9*) was observed in the microbial fermentation mixture-treated plants compared with untreated plants. These genes are often associated with the elicitation of plant defence responses to specific biotrophic pathogens, such as powdery mildew, suggesting an elicitor-mediated response in the wheat plants tested. The product components were assessed, and the components were found to act synergistically in the microbial fermentation mixture. Therefore, this fermentation-based elicitor provides an effective method for powdery mildew control.

## Introduction

It is conservatively estimated that fungal pathogens alone are responsible for losses of 15% to 20% of wheat production annually (Figueroa et al., 2018), costing billions of dollars to the global economy (Dean et al., 2012). The severity of disease caused by pathogens, including *Blumeria graminis* f. sp. *tritici*, the causal agent of wheat powdery mildew, increases with the intensity of production (Oerke, 2006). Yield losses caused by powdery mildew in wheat can range from 13% to 20% in winter and spring wheat, respectively (Griffey et al., 1993; Conner et al., 2003; Lackermann et al., 2011). Growers have traditionally relied on chemical fungicides to control these diseases, but as many fungal pathogens evolve resistance over time, new methods of control are needed to delay or impede the development of fungicide resistant pathogens. Finding efficient alternatives to chemical fungicides as part of an integrated pest management (IPM) system is of increasing importance particularly with regards to the EU Framework Directive 2009/128/EC on the sustainable use of pesticides.

The idea that an externally applied elicitor could improve crop fitness and induce plant defences was first investigated in the early 1900s (Chester, 1933). However, the first recognized study to verify this hypothesis came in the late 1970s, when applications of salicylic acid (SA) were reported to promote resistance against tobacco mosaic virus (TMV) while inducing the expression of pathogenesis-related (PR) genes (White, 1979). Preparing uninfected systemic tissues for a subsequent enhanced defence response to plant pathogens, is called systemic-acquired resistance (SAR) or “priming” (Bektas and Eulgem, 2015; Schwachtje et al., 2018).

Previous studies have been performed to test the efficacy of microbial fermentation products (MFPs) as elicitors of plant defence, including a yeast cell wall extract derived from the beer brewing process (Yaguchi et al., 2017), and a compound derived from glutamate fermentation (Chen et al., 2014). Derivatives of the yeast cell wall, including glucan, mannon, and chitin, have all been investigated as inducers of plant defence (Reglinski et al., 1994; Boller, 1995; Reglinski et al., 1995; Minami et al., 2011; Narusaka et al., 2015; Yaguchi et al., 2017), whereas lactic acid bacteria have been shown to prevent fungal disease in the field (Oliveira et al., 2014).

Most chemical fungicides have clearly defined active ingredients with specific modes of action. However, a number of the compounds used for plant priming today are from biological sources, often with more than one ingredient, and with that, defining a clear mode of action becomes more challenging. In some cases, product components may work independently, or have an additive or synergistic effects (Návarová et al., 2012; Sharma et al., 2014; Bernsdorff et al., 2015; Conrath et al., 2015; Saa et al., 2015; Yakhin et al., 2016) or in some cases be directly anti-fungal, inhibiting fungal development (Hansjakob et al., 2010; Nesler et al., 2015).

Among the reported plant responses induced are: the promotion of cell wall fortification or the boosting of a signaling pathway and the subsequent activation of SAR (Reignault et al., 2001; Návarová et al., 2012; Aranega-Bou et al., 2014). Enhancement of plant defence, stimulated by an external source, is also sometimes called induced resistance (IR) (Pastor et al., 2014) and several exogenously applied substances have been shown to induce plant resistance (Reignault et al., 2001; Li et al., 2016; Reimer-Michalski and Conrath, 2016). An inducible plant defence response is activated when a pathogen-associated molecular pattern (PAMP) or microbe-associated molecular pattern (MAMP) is recognized by the plants pattern recognition receptors (PRRs), triggering a multifaceted immune response resulting in increased disease resistance (Jones and Dangl, 2006; Bigeard et al., 2015). Bacterial molecules, for example, lipopolysaccharides and flagellin and fungal molecules, such as chitin, are PAMPs (Bigeard et al., 2015). When a plant has been stimulated into IR, but has not yet been challenged by a pathogen, it is in the priming phase (Pastor et al., 2014). Plants that have been primed react to a biotic or an abiotic stress and require only low levels of stimulus to initiate a defence response (Conrath, 2009). This resistance is mediated by one or more plant hormone signaling pathways. The SA signaling pathway is involved in local defence against an extensive range of biotrophic and hemi-biotrophic pathogens consequently triggering SAR (Huot et al., 2014; Bernsdorff et al., 2015). While jasmonic acid (JA) has also been implicated in powdery mildew defence in wheat (Duan et al., 2014). When a plant is stimulated with an elicitor, it triggers the priming state or phase where metabolites and other molecules are placed in a state of readiness. The plant remains primed until it encounters a stress event and then enters the post-challenge primed state, characterized by an accelerated response to mitigate that stress (Balmer et al., 2015).

The aim of this study is to assess if a fermentation derived elicitor mixture can provide resistance against powdery mildew in wheat. The mode of action of the fermentation-based product was investigated including any direct anti-fungal properties and *via* the induction of key defence gene priming.

## Materials and Methods

### Plant Material

Two commercial cultivars of wheat (*Triticum aestivum*) were used; cv. Avatar, which is a British cultivar currently on the recommended list in Ireland for 2019 with a resistance rating of 5 (moderately susceptible to powdery mildew) (DAFM, 2018) and cv. Remus, a German cv. from 1996 which had a resistance rating of 5 at that time (Planzenshutz, 1996). Seeds of these cultivars were removed from storage at 4°C, sterilized in 20% bleach for 4 min and rinsed in sterilized distilled water at least four times. To induce germination, seeds were incubated on moist filter paper in sealed petri dishes (15–20 seeds/dish) under sterile conditions in darkness at 24°C for 4 days and then transferred to pots of John Innes no. 2 soil, with no other nutritional amendments in a growth chamber under a 16-h day/8-h night photoperiod at 13,000 lux, RH 80% ± 5% and a constant temperature of 20°C and kept under these conditions for the full duration of the experiments.

### Elicitor Treatments

The test product is a liquid MFP, consisting of a proprietary blend containing bacteria and yeast from fermentation brewing media (Bokulich and Bamforth, 2013) and copper as copper sulphate (Alltech Crop Science, KY, USA). MFP will be provided on request. The product was prepared as per manufacturer’s recommendations of 0.5% V/V in sterile distilled water. A solution of MFP without the copper component was supplied which was also diluted 0.5% V/V in sterile distilled water. This solution is referred to as nC herein. The MFP stock solution contains 4.7% elemental copper in the form of CuSO_4_. Solutions of CuSO_4_ containing 4.7% elemental copper were made for experiments and diluted to 0.5% V/V in sterile distilled water. For experiments, a 4-ml volume of these solutions was foliar applied to each 3-week-old seedling. Sterilized distilled water was used as the control treatment.

### Disease Assays

To investigate if the product could impede powdery mildew pathogenesis, 3-week-old seedlings at the three leaf stage (10 plants per treatment for each cultivar) were treated with MFP and again 1 week later before inoculation with powdery mildew 1 day after the second treatment. To assess the priming effects of MFP treatment for disease assays, 3-week-old seedings (12 plants/treatment) were treated once, 8 days prior to the pathogen challenge. Disease severity was assessed by counting the number of pustules when pustules became visible and were still distinguishable as individual colonies (Reignault et al., 2001). Disease assays were used to investigate the components of MFP (CuSO4 and nC) on wheat following inoculation with *Bgt*. At 7 days post inoculation (dpi), the number of pustules/cm^2^ and the number of spores/cm^2^ were measured. For spore counts, the treated leaf tissue from each seedling was harvested and placed in a 15-ml falcon tube. A 1-ml volume of distilled water was added, and spores were liberated by vortexing for 30 s at 3000 rpm. Spores were counted in a hemocytometer and normalized to the leaf area (modified from Weßling and Panstruga, 2012).

### Fungal Isolate and Inoculation Procedure

**Figure 1:**
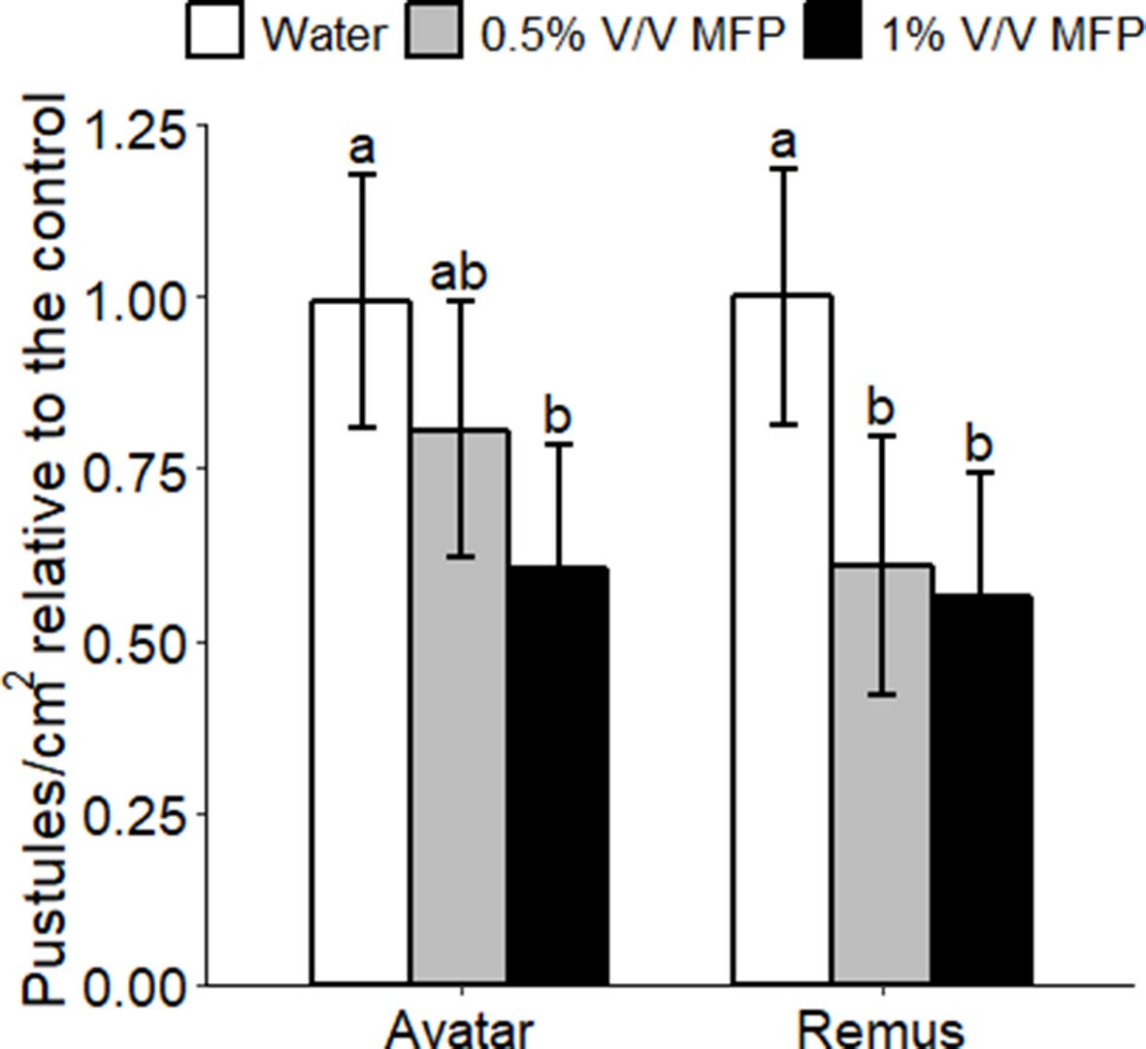
Effect of MFP against powdery mildew on wheat seedlings. Wheat seedlings received two MFP sprays 1 week apart and were inoculated with *Bgt* (inoculum ∼20 conidia per cm^2^) 1 day after the second treatment. Disease severity was assessed by counting the number of pustules/cm^2^, 7 days post inoculation (dpi). Disease severity was measured on wheat seedlings of cv. Avatar and Remus treated with water, 0.5% V/V MFP or 1% V/V MFP in a growth chamber. Results show pustules/cm^2^ relative to the control which were set to 1, bars represent the mean of three independent experiments where 10 plants were inoculated per cultivar and error bars indicate 95% confidence intervals. Different letters denote significant differences between treatments and cultivars (*p* < 0.05).

Inoculations were performed on seedlings using a settling tower method (Vogel and Somerville, 2000; Fraaije et al., 2002; Weßling and Panstruga, 2012). Wheat seedlings (20 pots with three seedlings per pot) in 7-cm^2^ pots were placed at the bottom of a 1.3-m-tall cardboard tower with a hemocytometer to determine spore densities (approximately 20 conidia per cm^2^ (**Figures 1** and **5**) or approximately 500 conidia per cm^2^ (**Figure 6**) (Randoux et al., 2006; Matić et al., 2018). One or 10 infected wheat seedlings were used as a source of inoculum by gently tapping the infected plant over the settling tower. The spores were allowed to settle for 20 min before removing the settling tower and infected plants moved to the growth chamber. The *B. graminis* f. sp. *tritici* isolate was collected from a Teagasc field site at Oakpark Carlow, Ireland in 2015 and a single spore isolate prepared as described by Korhonen and Hintikka (1980). The disease was increased and maintained on wheat seedlings in open transparent plastic bags at room temperature and under ambient light.

### *B. graminis* f. sp. *tritici* Germination Sensitivity Assay

The sensitivity of *B. graminis* f. sp. *tritici* conidia germination to increasing MFP concentrations was assessed *in vitro*. Fresh wheat cv. Avatar leaves (28 g/L) were macerated, added to distilled water, and brought to the boil. The solution was then stirred for 5 min. Cold distilled water was added to adjust to the correct volume. The solution was poured through filter paper. A 1.5% technical No. 2 agar was made from the solution and autoclaved (method modified from Morrison, 1964; Arabi and Jawhar, 2002 and Randoux et al., 2006). Serial dilutions of MFP were added, such that the following final rates in the agar would be obtained: 0.5% V/V, 0.34% V/V, and 0.17% V/V (the latter two concentrations being ⅔ and ⅓ the 0.5% V/V rate, respectively). Sterilized distilled water was used as a control. The development of 100 spores/plate was evaluated and germ tube development scored visually and recorded 48 h post inoculation (hpi).

### Real-Time Quantitative PCR Gene Expression Analysis

Three-week-old wheat seedlings were either treated with MFP and inoculated simultaneously or treated 8 days prior to inoculation. Tissue samples were taken at time points pertaining to certain developmental stages of the disease. A total of 100-mg leaf tissue was sampled, flash frozen in liquid N2, and stored at −80°C for RNA extraction. RNA was extracted using a RNeasy plant mini kit (Qiagen). An on-column DNase digestion (Sigma-Aldrich) was incorporated to eliminate genomic DNA from the total RNA preparations. An Omniscript^®^ Reverse Transcription Kit (Qiagen) was used to synthesize cDNA from the extracted RNA samples. This cDNA was used for PCR and qPCR to analyze defence-related gene expression.

Defence-related genes were selected from the literature, and a shortlist was compiled based on the results of semi-quantitative PCR assays (**Supplementary Fig 1** and **Table S1**). Primer sequences used in qPCR experiments are shown in **Table S1**. Four leaves, two from each of two plants were pooled as a biological sample. For analysis of concurrent treatment and inoculation experiments, three technical replicates were performed for each template and primer combination. This was repeated in four independent experiments. For analysis of the 8-day pre-treatment experiments, four technical replicates were performed for each template and primer combination. This was repeated in three independent experiments. Relative gene expression was calculated with the equation 2^-(Ct target gene – Ct housekeeping gene)^ using the qRT-PCR threshold cycle (Ct) values. qPCR analysis of concurrent treatment and inoculation experiments were performed on a Stratagene Mx3000p and analysis of the 8-day pre-treatment experiments were performed on an Applied Biosystems QuantStudio 7flex.

### Statistical Analysis

Data were analyzed using the R statistical package (https://www.r-project.org/). Statistical analysis of disease assays investigating powdery mildew pathogenesis and evaluating the reduction of disease symptoms on pre-treated plants analysis of variance (ANOVA) and Tukey’s test was performed. Disease assays to investigate the effect of each component of MFP (CuSO_4_ and nC) against *B. graminis* f. sp. *tritici*, were analyzed using a zero-inflated regression model. Analysis of qPCR data was performed using a general linear model. Data were log transformed prior to analysis. Proportion data from *in vitro* experiments were analyzed using generalized linear models with a quasi-binomial distribution. Figures were produced using the R statistical package and GraphPad Prism version 6 for Windows, (GraphPad Software, San Diego, CA).

## Results

### Powdery Mildew Pathogenesis

To test whether the MFP could protect against powdery mildew in wheat, using the recommended rate of application or double the rate, Avatar or Remus seedlings were treated twice with MFP at either 0.5% V/V or 1% V/V rate. Plants received two MFP treatments, at a week apart, and were inoculated with powdery mildew conidia (*B. graminis* f. sp. *tritici*, *Bgt*) 1 day after the second treatment. Powdery mildew disease severity on wheat plants was significantly reduced by pre-treatment with MFP. An application of 1% V/V significantly reduced powdery mildew symptoms on both cultivars, by an average of 44%, on the susceptible cultivar Remus (*p* = 0.014) and by 39% on the moderately susceptible cultivar Avatar (*p* = 0.039) compared to water-treated control (**Figure 1**). An application of 0.5% V/V significantly reduced symptoms only on Remus (by 38%, *p* = 0.046) (**Figure 1**). We sought to use a relevant commercial variety and therefore selected Avatar which is currently on the recommended list in Ireland (DAFM, 2018) (resistance rating of 5) for subsequent experimental work.

### *In Vitro* Powdery Mildew Conidia Germination on Agar Amended With MFP

**Figure 2:**
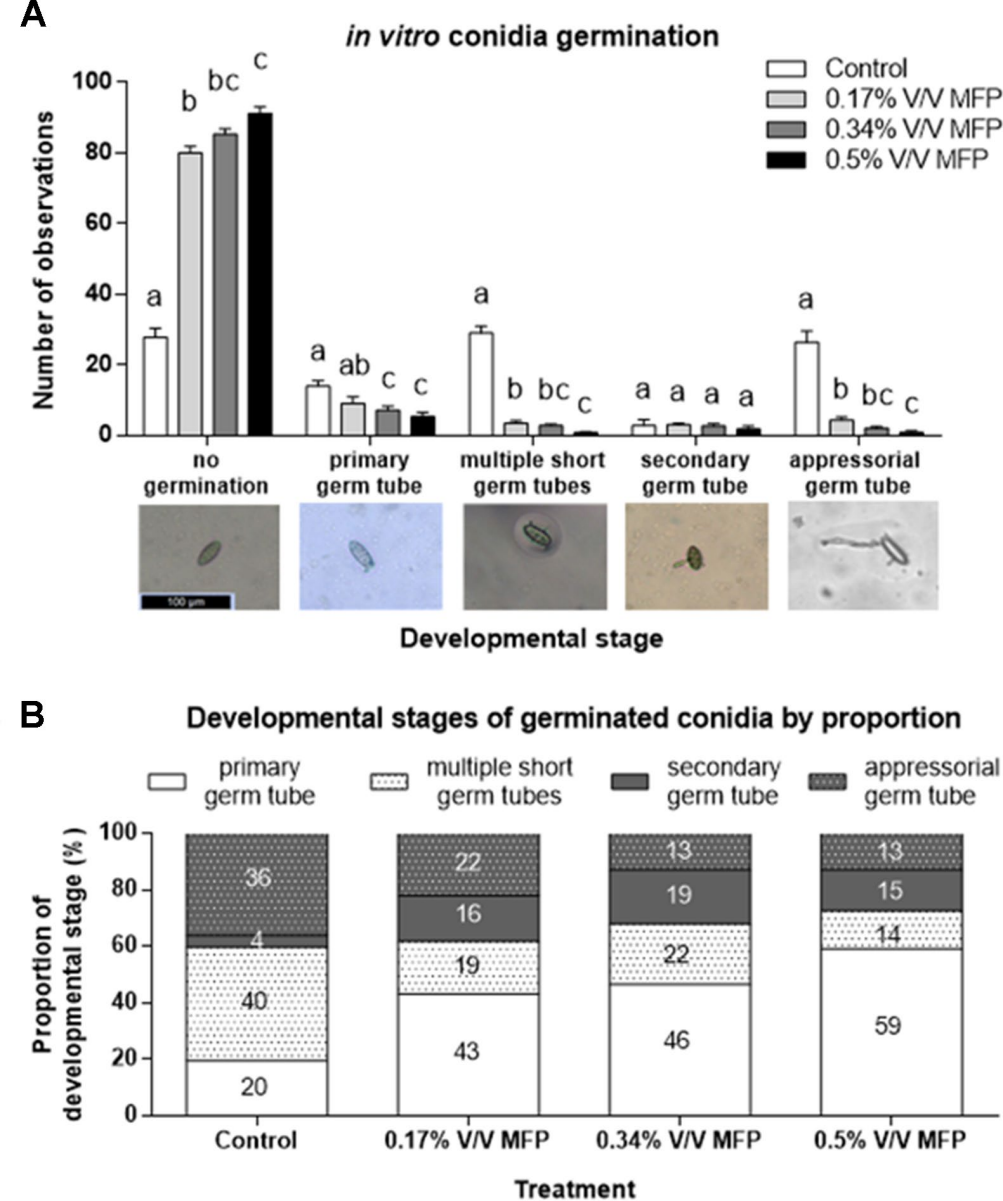
Assessment of the sensitivity of *Blumeria graminis* f. sp. *tritici* conidia germination to increasing concentrations of MFP. **(A)** Three product concentrations were assessed. 0.5% V/V MFP, 0.34% V/V MFP, and 0.17% V/V MFP V (the latter two concentrations being ⅔ and ⅓ the 0.5% V/V rate, respectively) on agar supplemented with wheat leaf extract. Sterilized distilled water was used as a control. The development of 100 spores/plate of three plates/treatment in each independent experiment was evaluated and recorded. Results represent the mean and error bars indicate SEM of three independent experiments (n = 900). Different letters denote significant differences between treatments (*p* < 0.05). Categories of pre-penetration development recorded were: no germination, presence of primary germ tube, presence of multiple short germ tubes, presence of secondary germ tube, and presence of differentiated appressorial germ tube. **(B)** Proportional representation of developmental stages on germinated spores. Numbers represent percentages.

The effects of MFP on the germination of *B. graminis* f. sp. *tritici* conidia was determined *in vitro* by growing the pathogen in media supplemented with wheat leaf extract and MFP at three different concentrations; 0.5% V/V, 0.34% V/V, and 0.17% V/V. For the concentrations tested, the inhibition of spore germination increased as the concentration of MFP increased. Significantly more non-germinated spores were recorded on all MFP treatments compared with the control (*p* ≤ 0.001, **Figure 2A**). There were also significantly less differentiated appressorial germ tubes visible on any of the MFP treatments compared with the control (*p* ≤0.001, **Figure 2A**).

A subset of the data was analyzed excluding non-germinated spores to assess if MFP affects *Bgt* development stage post germination (**Figure 2B**). The proportion of germinated conidia with only one primary germ tube was significantly higher on 0.17% V/V (*p* = 0.03), 0.34% V/V (*p* = 0.01), and 0.5% V/V MFP (*p* < 0.001) plates compared with control plates (**Figure 2B**). This was an increase of 23%, 27%, and 40%, respectively, from 20% on the control plates. Conversely, the proportion of germinated conidia with multiple primary germ tubes was significantly lower on 0.17% V/V (*p* = 0.03) and 0.5% V/V MFP (*p* = 0.004) plates, a reduction of 21% and 26% respectively from 40% on the control plates. The proportion of germinated conidia with appressorial germ tubes was also significantly lower on 0.34% V/V (*p* = 0.01), and 0.5% V/V MFP (*p* = 0.01) plates, a 23% decrease for both MFP concentrations from 36% on the controls. Therefore, the inhibition of *Bgt* germ tube development increased as the concentration of MFP increased.

### Wheat Defence-Related Genes Induced by MFP

**Figure 3:**
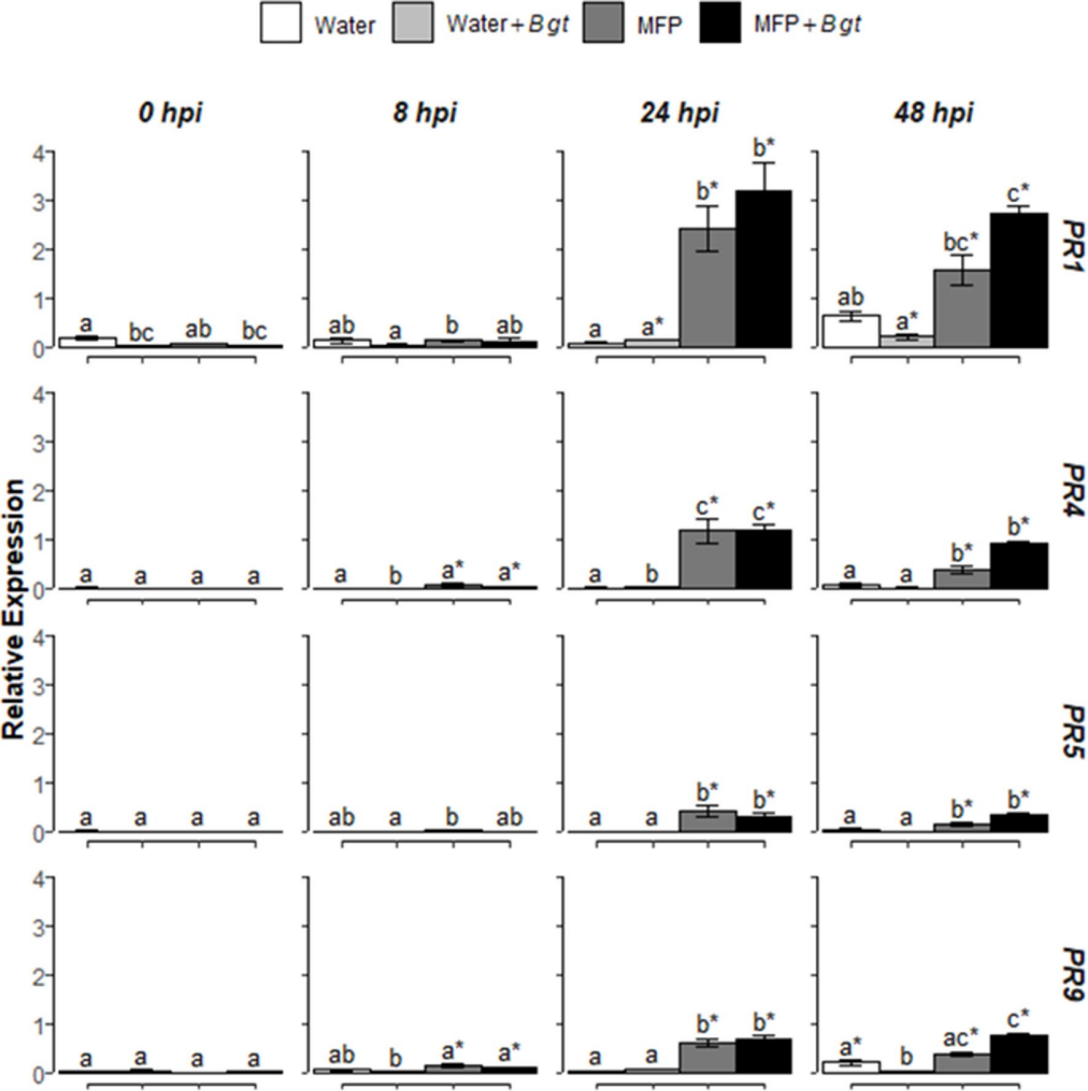
Effect of concurrent MFP treatment and inoculation with *Blumeria graminis* f. sp. *tritici* (Bgt), on defence gene expression in wheat leaves (cv. Avatar). Shown are *PR1*, *PR4*, *PR5*, and *PR9* expression values at 0, 8, 24, and 48 h post inoculation (hpi). The wheat alpha-tubulin gene was used as internal reference to calculate the relative expression of *PR1*, *PR4*, *PR5*, and *PR9* using the equation 2^-(Ct target gene – Ct housekeeping gene)^. Wheat leaves were treated with MFP or water and immediately inoculated with *B. graminis* f. sp. *tritici*. The tissues were harvested at various time points as indicated. Results represent the mean (two leaves from two plants pooled as a biological sample with three technical replicates, repeated in four independent experiments) and error bars indicate ± SEM. Different letters denote significant (*p* ≤ 0.05) differences between treatments per time point. Asterisk denotes significant (*p* ≤ 0.05) difference of treatment at later time points compared to 0 hpi.

To investigate if MFP would induce defence genes and if resistance induction in wheat was a factor for reduced disease severity (**Figure 1**), the relative expression levels of four defence-related genes were analyzed using RT-qPCR (**Figure 3**). Various SA, JA, and ROS defence genes were also investigated using RT-PCR but only *PR1*, *PR4*, *PR5*, and *PR9* were consistently upregulated by MFP (**Supplementary Fig 1** and **Table S1**). Therefore, these genes were selected for further study. Wheat seedlings were treated with MFP or water and immediately inoculated with powdery mildew conidia (*Bgt*). Transcript levels of *PR1*, *PR4*, *PR5*, and *PR9* were quantified at 0, 8, 24, and 48 hpi. Inoculation of water-treated plants with *Bgt* did not significantly induce the expression of these genes to above basal levels at any of the timepoints measured. However, at 24 hpi, MFP treatment and MFP inoculated with *Bgt* (MFP+*Bgt*) induced significantly higher transcript levels of *PR1*, *PR4*, *PR5*, and *PR9* compared with the water control, but there was no statistical difference between the two (MFP and MFP+*Bgt*).

### Eliciting Gene Expression—Post-Biotic Challenge, on Tissue Pre-Treated With MFP

**Figure 4:**
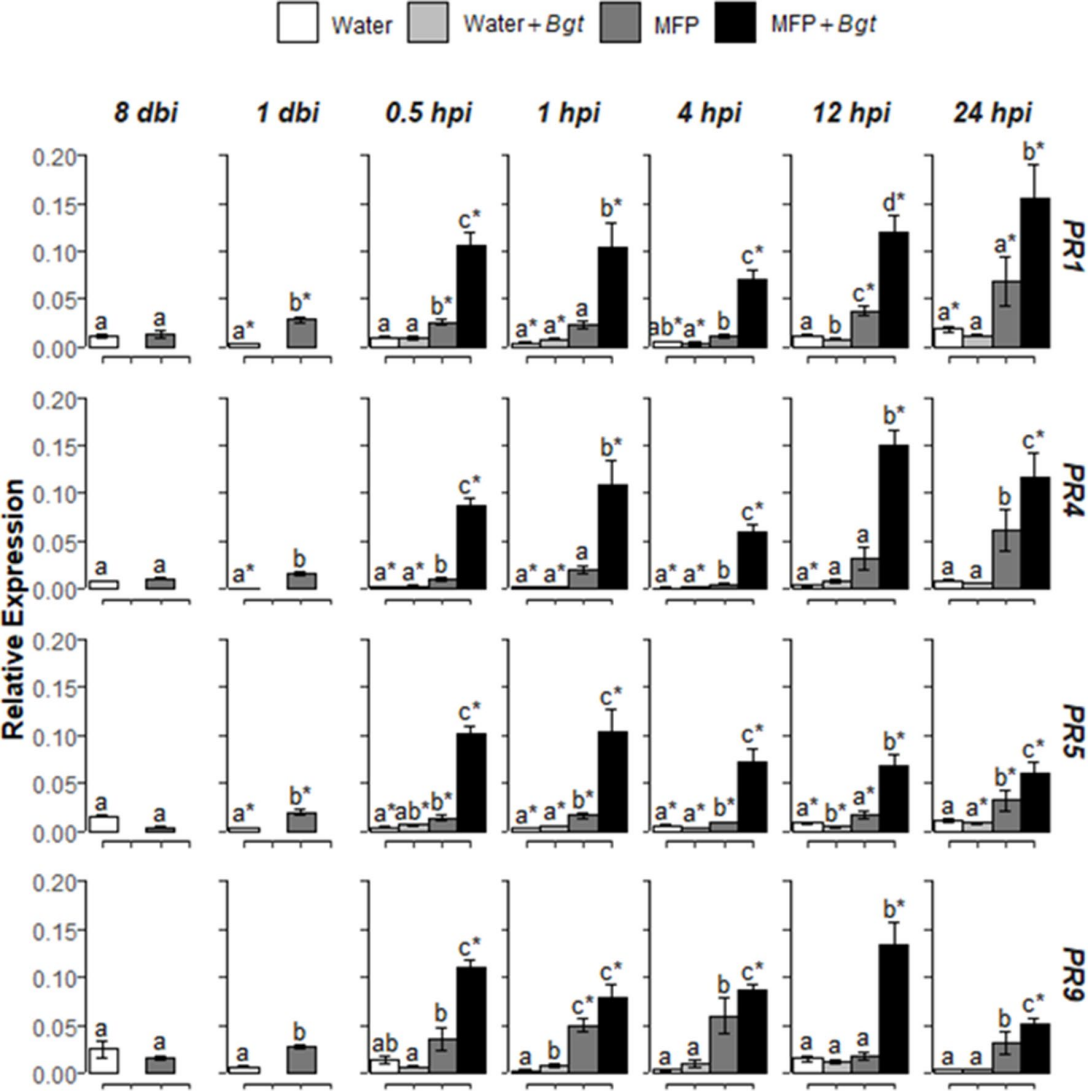
Effect of MFP treatment on defence gene priming. Shown are *PR1*, *PR4*, *PR5*, and *PR9* expression values at 8 days before inoculation (dbi), 1 dbi and 0.5, 1, 4, 12, and 24 h post inoculation (hpi) with *Blumeria graminis* f. sp. *tritici* on inoculated MFP-treated and water-treated plants compared to control plants. The wheat alpha-tubulin gene was used as internal reference to calculate the relative expression of *PR1*, *PR4*, *PR5*, and *PR9* using the equation 2^−(Ct target gene – Ct housekeeping gene)^. Results represent the mean (two leaves from two plants pooled as a biological sample with four technical replicates, repeated in three independent experiments) and error bars indicate ± SEM. Different letters denote significant (*p* ≤ 0.05) differences between treatments per time point. Asterisk denotes significant (*p* ≤ 0.05) difference of treatment at later time points compared to 0 hpi.

Subsequently, MFP was tested as a priming agent of defence genes in wheat plants against powdery mildew. Treatment of MFP was applied 8 days prior to inoculation with *B. graminis f*. sp. *tritici(Bgt)* pathogen inoculation (**Figure 4**). No significantly higher expression was found for *Bgt* inoculated with water-treated tissue (Water + *Bgt*) with the exception of *PR9* at 1 hpi (*p* = 0.002). On MFP-treated tissue, transcripts of the four *PR* genes were significantly induced, in the majority of timepoints post inoculation. Within 30 min (at 0.5 hpi) of inoculation, all four genes showed significantly higher expression on MFP + *Bgt* compared with MFP only and water treatments (*p* ≤ 0.05). This was found across all timepoints, with one exception of 1 hpi, where expression of *PR9* was statistically similar to that induced by MFP (*p* = 0.627).

### Evaluating Reduction of Disease Symptoms on Pre-Treated Plants

**Figure 5:**
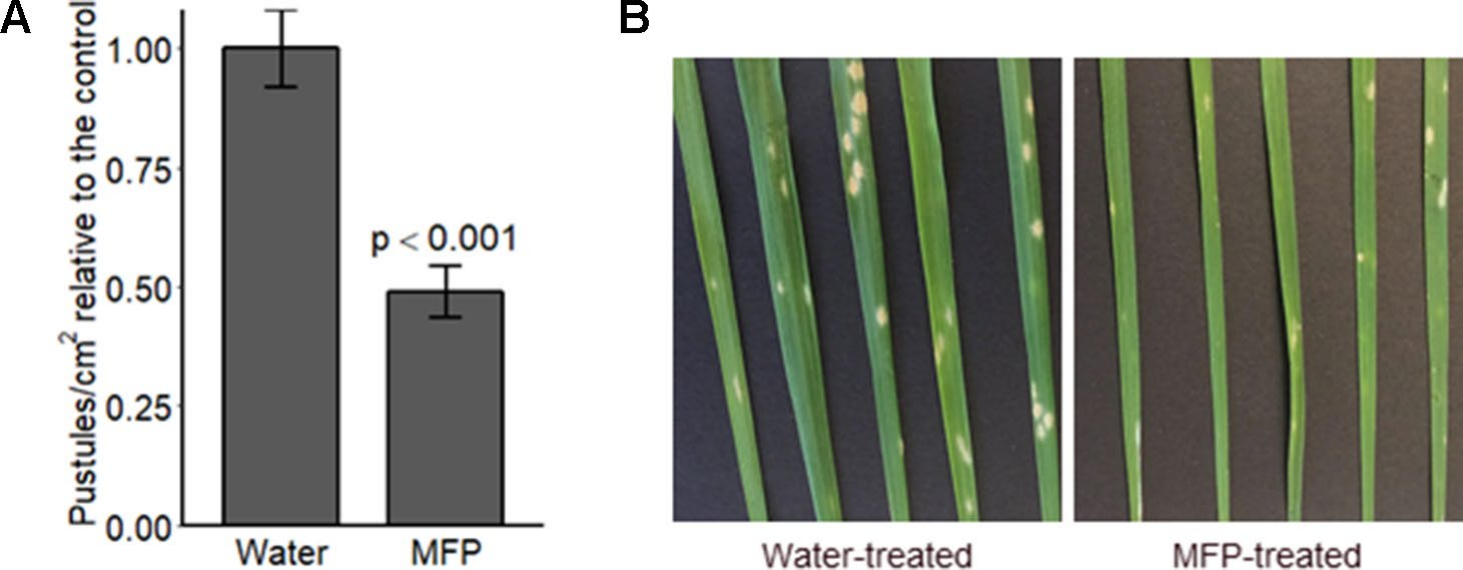
Disease level on wheat seedlings cv. Avatar pre-treated with MFP, 8 days prior to inoculation with powdery mildew. Wheat seedlings received one MFP spray and were inoculated with *Bgt* 8 days later (inoculum ∼20 conidia per cm^2^) **(A)** Disease severity was assessed by counting the number of pustules/cm^2^, 7 days post inoculation (dpi). Results show pustules/cm^2^ relative to the control which were set to 1, bars represent the mean of three independent experiments where 12 plants were inoculated, and error bars indicate 95% confidence intervals (n = 36). **(B)** Representative leaf symptoms.

We sought to investigate if priming specifically would provide protection to mildew. Therefore, *Bgt* conidia were applied 8 days after MFP treatment this was to allow defence gene expression levels (**Figure 4**) to return to basal levels before pathogen challenge (Balmer et al., 2015). Disease assays were performed to investigate if a reduction in disease symptoms occurred on plants primed with MFP 8 days prior to *Bgt* inoculation. Powdery mildew pustules/cm^2^ were significantly reduced, on MFP-treated leaves, an average of 51.2% lower than on water treated (**Figure 5A**, *p* < 0.001). Though symptoms were reduced, pustules that had developed were at the same developmental stage as those on the water-treated plants (**Figure 5B**).

### Evaluating MFP Components—Post Biotic Challenge on Tissue Pre-Treated With the Copper and Non-Copper Component of MFP

**Figure 6:**
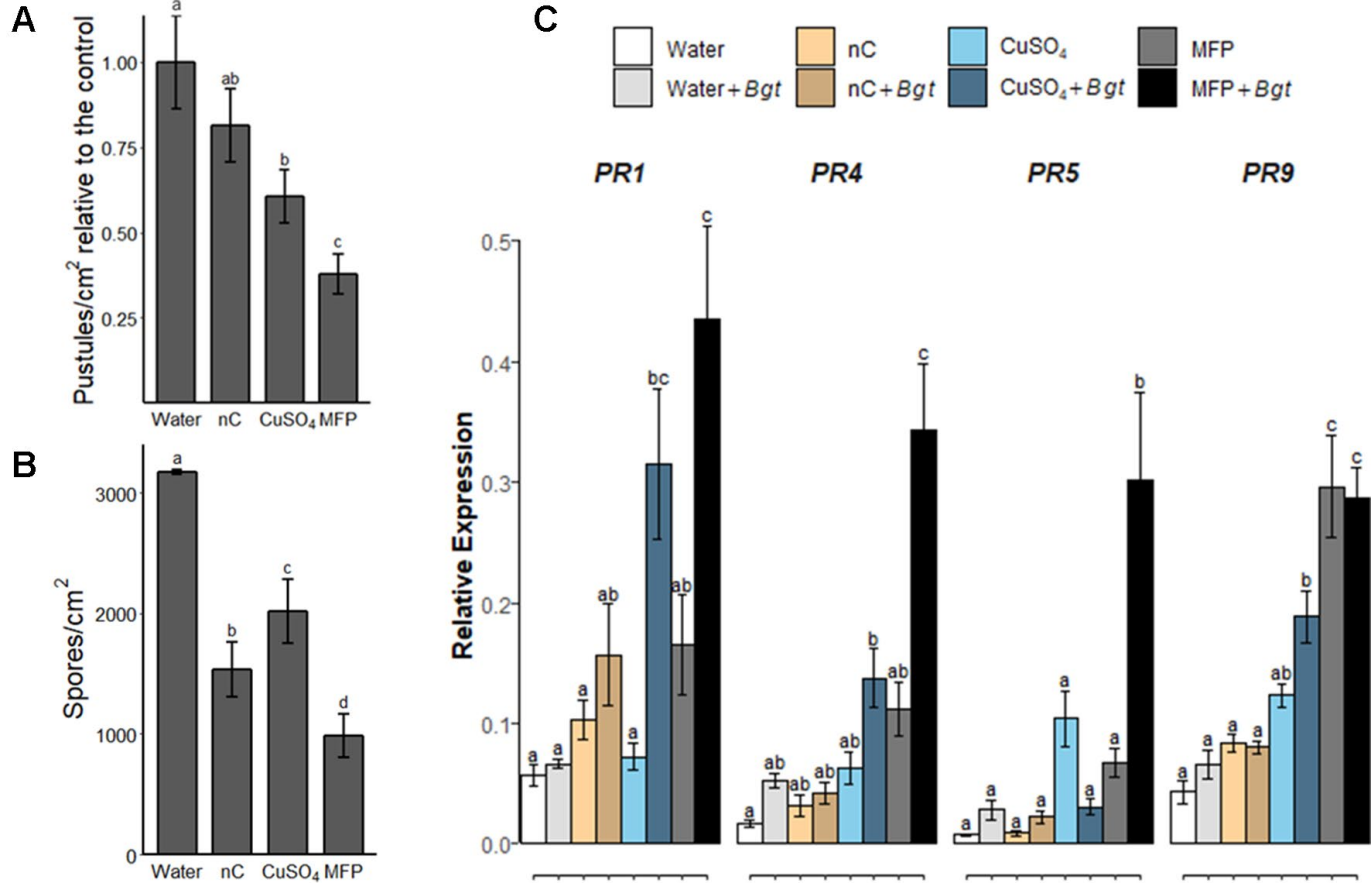
Effect of copper and non-copper component of MFP treatment on powdery mildew disease levels and defence gene priming. Wheat seedlings cv. Avatar were pre-treated with water, MFP, CuSO_4_, or nC, 8 days prior to inoculation with *Bgt* (inoculum ∼500 conidia per cm^2^). **(A)** Disease severity was assessed 7 dpi by counting the number of pustules/cm^2^. Results show pustules/cm^2^ relative to the control which were set to 1, bars represent the mean of three independent experiments and error bars indicate SEM (n = 30). Different letters denote significant (*p* ≤ 0.05) differences between treatments. **(B)** Analysis of spore counts at 7 dpi, treated leaf tissue samples were harvested and spores were liberated by vortexing in distilled water. Spores were counted in a hemocytometer and normalized to the leaf area. Bars represent the mean of three independent experiments and error bars indicate 95% confidence intervals (n = 30). Different letters denote significant (*p* ≤ 0.05) differences between treatments. **(C)** Induction of defence gene expression at 0.5 h post inoculation (hpi), inoculated with *Bgt* on CuSO_4_-treated, nC-treated (nC = non-copper part of MFP), MFP-treated and water-treated plants compared to control plants. The wheat alpha-tubulin gene was used as internal reference to calculate the relative expression of *PR1*, *PR4*, *PR5*, and *PR9* using the equation 2^-(Ct target gene – Ct housekeeping gene)^. Results represent the mean (two leaves from two plants pooled as a biological sample with four technical replicates, repeated in three independent experiments) and error bars indicate ± SEM. Different letters denote significant (*p* ≤ 0.05) differences between treatments.

To investigate the contribution of the MFP components towards powdery mildew control, wheat plants were treated with water, MFP, CuSO_4_, or MFP excluding the copper component (herein referred to as nC) (**Figure 6**). Eight days later, plants were inoculated with *Bgt* and at 7 days post inoculation pustules/cm^2^ were measured (**Figure 6A**). A higher inoculum level was also used (approximately 500 conidia per cm^2^ versus 20 conidia per cm^2^) to assess the efficacy of these components at a higher disease pressure. Seven days post *Bgt* inoculation both MFP- and CuSO4-treated leaves had significantly lower pustules/cm^2^ than water treated (*p* <0.0001, *p* = 0.001 respectively). However, MFP afforded the highest level of control and had significantly lower pustules/cm^2^ than CuSO4 (*p* = 0.035). *Bgt* spore production was also assessed; significantly less spores/cm^2^ were produced on leaves following nC, CuSO4, and MFP (*p* < 0.0001) treatments compared with water. MFP treatment also led to the lowest number of *Bgt* spores produced per cm^2^, and this was significantly less than nC (*p* < 0.001) and CuSO4 (*p* < 0.0001) (**Figure 6B**).

To investigate the contribution of the MFP components to priming; the priming effect of MFP, CuSO_4_, and nC was evaluated in wheat plants against powdery mildew (**Figure 6C**). Treatment of CuSO_4_, nC or MFP was applied 8 days prior to inoculation with *Bgt* pathogen inoculation as above. At 0.5 hpi, no significant expression of any of the tested genes was observed following either nC-, nC+*Bgt*-, or Water+*Bgt*-treated leaves. All four *PR* genes were significantly induced by MFP+*Bgt* compared to Water+*Bgt*. Induction of *PR9* was also observed on MFP-treated leaves. Three genes, *PR1*, *PR4*, and *PR9* were significantly induced by CuSO4+*Bgt* compared with Water+*Bgt*. However, the induction of *PR4*, *PR5*, and *PR9* observed on MFP+*Bgt* was significantly higher than that of CuSO4+*Bgt* alone.

## Discussion

In the present study, evidence is provided suggesting MFP impacts on the interaction between wheat and *B. graminis* f.sp. *tritici* on two fronts. First, *via* a direct antifungal action inhibiting conidia germination and germ tube differentiation, and second, by priming and enabling the induction of plant defences in response to a pathogenic challenge. Previously, Narusaka et al. (2015) found that a yeast cell wall extract had no direct antifungal or antibacterial action. Here we found that at low concentrations (0.17% V/V), MFP significantly reduced conidia germination (**Figure 2A**). The proportion of germinated conidia with multiple primary germ tubes or with appressorial germ tubes was also significantly reduced. In the present study, the antifungal action shown *in vitro* may be due, at least in part, to the copper sulphate component of MFP. Though the copper content is very low (an application rate of 0.02% compared to typically used rates of ≥0.2% (Arya and Perelló, 2010), a reduced copper dose complexed to peptides and amino acids is believed to facilitate greater penetration into pathogenic cells (Pertot et al., 2006; Malachová et al., 2011).

A reduction in powdery mildew disease symptoms was observed on wheat plants treated with the MFP. On cv. Remus which is an older cultivar from 1996 (Planzenshutz, 1996) and cv. Avatar which is currently on the Irish recommended list (DAFM, 2018), the reduction of disease symptoms following pre-treatment with 1% V/V MFP was found to be similar (**Figure 1**) suggesting the defence pathways activated were conserved in both varieties. This is possible since both varieties had a resistance rating of 5. Although, a low inoculum rate (of ∼20 conidia per cm^2^) was used for this initial assessment of MFP efficacy for powdery mildew protection, subsequent disease assays with MFP and components at a higher inoculum rate (∼500 conidia per cm^2^) also reduced powdery mildew susceptibility (**Figure 6A**). A previous study showed that a fermentation by-product supplemented with micronutrients caused a reduction of disease symptoms with the bacterial pathogen *Pseudomonas syringae* pv. *maculicola* in the model plant *Arabidopsis*. Where induction of *PR1*, *PR2 PR4*, and *PR9* in *Arabidopsis* were found between 5 and 24 h post application of the glutamate fermentation by-product (Igarashi et al., 2010). Although the fermentation by-product tested by Chen et al. (2014) induced defence genes, including stilbene synthase, the chitinase *CHIT-4c*, and *PR2* (b-1,3-glucanase) in cultured grape cells. An activator derived from the yeast cell wall also induced expression of *PR1* in *Arabidopsis*, which peaked 1 day post treatment (Narusaka et al., 2015). A strong activation of *PR* transcripts was recorded in this study following MFP treatment, analyzed using qPCR. Plant *PR* proteins are anti-microbial and have well-established functions in plant defence during pathogen attack (Desmond et al., 2005; Desmond et al., 2008). Recently, PR-1 family members have been demonstrated to bind sterols (Breen et al., 2017). *PR4* (wheatwin) which encodes an endochitinase is involved in the degradation of fungal cell wall chitin (Desmond et al., 2005). It has been reported to be induced by wounding, through activators of SAR and by *Fusarium* pathogens (Desmond et al., 2008). *PR5* was also induced by MFP, which exhibits antifungal activity (Li et al., 2015) and was reported to be activated by exogenous methyl jasmonate in wheat (Duan et al., 2014). Finally, *PR9*, which is a peroxidase with a role in ROS production and plant cell wall formation and lignification (Casassola et al., 2015) was induced. All four *PR* genes were previously implicated in variety specific powdery mildew resistance in wheat (Duan et al., 2014).

In addition to *PR* gene activation, a yeast cell wall extract added to rice cell suspension culture induced accumulation of signaling molecules involved in SAR; azelaic acid (AzA) and phenylalanine (Phe), as well as the wound-based signaling compound JA, and related jasmonate intermediates and actives 12-hydroxyjasmonoyl isoleucine and 12-oxo-phytodienoic acid (Yaguchi et al., 2017). These compounds were proposed to prime against the necrotrophic pathogen *Botrytis cinerea* in *Arabidopsis*. Although the MFP tested here includes yeast cell wall, we did not observe induction of the allene oxide synthase (AOS) which is involved in JA biosynthesis (**Supplementary Fig 1** and **Table S1**). However, the biotrophic pathogen *Bgt* was used in this study which may ultimately influence the defence genes induced following priming.

Copper has also been found to activate defence-related responses in plants. Copper chloride induced the activation of *PR1* in *Brassica carinata* 10 h after treatment, and CuSO4 induced the activation of defence-related genes, including *PR1* in pepper, 4 days post treatment (Zheng et al., 2001; Chmielowska et al., 2010). This suggests that the defence gene induction observed may also be influenced by the copper component of the MFP. Although, pre-treatment with CuSO_4_ did cause a rapid activation of *PR* defence genes upon *Bgt* inoculation, *PR4*, *PR5*, and *PR9* were significantly higher following *Bgt* inoculation in MFP-treated leaves. This indicates that the components of MFP are more effective at priming when combined than when applied individually (**Figure 6C**). Furthermore, assessment of the MFP components separately, CuSO4 and the microbial component (nC) in the disease assays (**Figures 6A, B**), also suggests that there is a synergistic effect between the priming mediated by nC and copper component since the MFP combination gives the greatest level of powdery mildew disease control (**Figures 6A, B**). Previous studies have observed such a synergistic effect with copper. For example, greenhouse trials investigating the synergism of copper fungicides and salicylaldehyde benzoylhydrazone (SBH) demonstrated that the copper component alone was moderately active, and SBH was weekly active, but that the combination delivered a significant reduction in disease caused by the wheat pathogen *Puccinia recondita* (Young et al., 2016)

When MFP was used here as a pre-treatment, it was found to prime wheat against powdery mildew (**Figure 4**). Over time, expression levels of *PR1*, *PR4*, *PR5*, and *PR9* induced by MFP treatment subsided and from 7 days post treatment, transcripts of *PR4* and *PR9* had returned to statistically comparable levels as the water-treated tissue, although *PR1* and *PR5* remained significantly higher (**Figure 4**). The period after application of a priming stimulus and prior to pathogen challenge is referred to as the priming phase (Balmer et al., 2015). During this phase, it is proposed that acetylation of histones occurs, enabling faster gene induction when subsequently challenged by a pathogen (Aranega-Bou et al., 2014). It has also been proposed that during the priming phase, the plant accumulates inactive protein kinases, and inactive defence metabolites that could be quickly utilized upon a pathogen challenge (Pastor et al., 2014). On MFP-treated tissue, all four *PR* genes were activated rapidly just 30 min (0.5 hpi) post inoculation with *Bgt* and remained expressed at least until 24 hpi (**Figure, 4**). At all post challenge timepoints, expression levels were also significantly higher than levels on non-*Bgt* inoculated MFP-treated tissue. This accelerated activation of defence genes post biotic challenge is consistent with the post challenge priming phase described elsewhere (Gamir et al., 2014; Pastor et al., 2014; Balmer et al., 2015). Other studies have found similar responses to fermentation-derived plant activators, for example, Narusaka et al. (2015) reported *PR1* expression at 10 hpi after inoculation with *P. syringae* pv. *maculicola* on *Arabidopsis* plants pre-treated with a yeast extract-based elicitor, although *PR* gene expression was not recorded as early as 30 min post inoculation as observed here on pre-treated wheat plants.

Yeast- and bacterial-based elicitors are likely to induce priming *via* PTI (Moon et al., 2015; Saijo et al., 2018). *B. graminis* f. sp. *tritici* is a well-adapted pathogen and in a compatible reaction initiates suppression of pathogen triggered immunity (PTI) *via* the production and secretion of effector proteins into plant cells (Bektas and Eulgem, 2015; Menardo et al., 2017). Activation of a defence response suggests that in MFP-treated tissue, *B. graminis* f. sp. *tritici* may be recognized as a potential threat *via* PTI. Although the mechanism for this recognition is still largely undefined. One explanation is that priming *via* MFP allows *Bgt* to be detected, *via* a MAMP response and the plant activates an accelerated response to the pathogen challenge, helping to impede pathogen colonization without subsequent suppression of defence *via Bgt* effectors. This is supported by the difference between the water+*Bgt* and MFP+*Bgt* treatments where defence gene expression is only induced in the latter (with the exception of *PR9* at 1phi). Disease assays (**Figure 5**) on plants that were treated with MFP 8 days prior to inoculation had significantly reduced disease symptoms (*p* < 0.001). MFP treatment caused a reduction in disease symptoms of 51% compared with water. This is in contrast to where plants received two treatments, at 8 days and 1 day before inoculation (**Figure 1**). In this case, disease reduction was less pronounced. It is possible that in the case of two MFP applications 1 week apart followed by *Bgt* inoculation 1 day later, that the first MFP treatment primed the wheat plants (priming phase), and that the second acted as an elicitor rapidly inducing defence genes prior to *Bgt* inoculation so that the wheat seedlings were already in the post challenge primed stated. Therefore, this requires further investigation to compare defence induction following a second MFP treatment to the same treatment with *Bgt* inoculation, to assess how often MFP application is required. In conclusion, these results suggest that the reduced susceptibility to powdery mildew in wheat is afforded as a result of a dual action, a direct antifungal response and the ability to prime the plants own defence systems to impede fungal colonization. These results support further investigation into the mechanism of action of MFP and its viability to be used to protect from powdery mildew disease in wheat. These findings support the potential application of products that contain biological elicitors and suggest that synergistic and improved effects may be gained in mixed products for crop production.

## Data Availability Statement

The datasets generated for this study are available on request to the corresponding author.

## Author Contributions

TT, MG and AF designed the experiments. TT performed the experiments. AF supervised the project. TT wrote the manuscript, AF and MG revised the manuscript.

## Funding

This work was supported by Alltech Crop Science.

## Conflict of Interest

The research was funded by Alltech Crop Science (Ireland) who also provided the MFP. MG was employed by the company Alltech.

The remaining authors declare that the research was conducted in the absence of any commercial or financial relationships that could be construed as a potential conflict of interest. Collection of data, analysis, and interpretation was carried out independently of Alltech. The authors have the full data set available.
