# Supplementary material for: A Microbial Fermentation Mixture Primes for Resistance Against Powdery Mildew in Wheat
Source: Front Plant Sci. 2019 Oct 9;10:1241. doi: 10.3389/fpls.2019.01241 (PMC6794463; doi:10.3389/fpls.2019.01241)
Supplement: Supplementary file 1 [file Table_1.docx]

**Table S1. Accession numbers, annotation and primers of genes investigated.**

| GenBank Accession No. *Gene* | Annotation | Primer sequence  (Forward and Reverse) | Reference |
| --- | --- | --- | --- |
| AY196004 –  *AOS* | Allene oxide synthase, a defence-related gene involved in the regulation of jasmonic acid biosynthesis. | ACCGTGTTCAACAGCTACGG  AGCGCCTCTATCGTCACCTT | (Gaudet *et al.*, 2011) |
| BQ161624 | A phytoalexin, agmatine coumaroyl transferase. | TGTAGCAGCAGTTCTTGAAAGTGTTC  CTCATCCTGCTACCGTCCTTCTT | (Desmond *et al.*, 2008) |
| BQ165963 | Peroxidase involved in the metabolism of reactive oxygen species (ROS). | CTATGAGCATCTCCGAAAGACGTT  CATACCGTTGGTGCGTACTACACTAC | (Desmond *et al.*, 2008) |
| CA667447.1 | A germin-like protein involved in the metabolism of reactive oxygen species (ROS). | GCTGAACTGCGAGGCTAACTTATT  TCCACAGAAGACATGTATTCAAGCT | (Desmond *et al.*, 2008) |
| CA682712 | Encodes a Flavonoid 7-O-methyltransferase-like protein involved in phytoalexin metabolism. | GACAACAAGGAGGCTGTGTATGG  GGTGTAATGCAGTTGAATCAAGGA | (Desmond *et al.*, 2008) |
| CD863039 | Encodes anti-microbial thaumatin-like proteins. | AGGTAATTTTTTTATTGCCCTGTACTG  TTACAGCCGCCGTACTACATGT | (Desmond *et al.*, 2008) |
| CK205943 | Encodes chitinase. | CATAAGGCGTGGATACACTGTCTAACT  CATGCGCTACTGCAGCATACTC | (Desmond *et al.*, 2008) |
| CK153727 –  *NPR3* | NPR1-like protein 3 gene. Anti-microbial. Key positive regulator, induced by SA, controlling the onset of SAR. | AAGCCTGTCACATGTCAAACC  GGGAAGCTAGCAAGGTTTTGT | (Gaudet *et al.*, 2011) |
| AF384143.1 –  *PR1* | Pathogenesis-related 1, β-1,3-glucanases are involved in stress response and plant defence and play a role in the regulation of callose deposition and in the hydrolysis of fungal cell wall. | CAATAACCTCGGCGTCTTCATCAC  TTATTTACTCGCTCGGTCCCTCTG | (Casassola *et al.*, 2015) |
| AB029934 –  *PR3* | Pathogenesis-related 3, possible involvement in JA mediated resistance in wheat. | AGAGATAAGCAAGGCCACGTC  GGTTGCTCACCAGGTCCTTC | (Duan *et al.*, 2014) |
| AF092123.1 –  *PR4* | Pathogenesis-related 4, endochitinase, these proteins hydrolyse chitin in fungal cell walls. | AAGTGCCTCCAGGTGACGAA  TGCACTGGTCGACGATCCT | (Casassola *et al.*, 2015) |
| AF442967 -  *PR5* | Pathogenesis-related 5. Thaumatin-like protein. | ACAGCTACGCCAAGGACGAC  CGCGTCCTAATCTAAGGGCAG | (Duan *et al.*, 2014) |
| AK331482.1 –  *PR9* | Pathogenesis-related 9. Peroxidase. Peroxidases are involved in many physiological and developmental processes, from germination to senescence, having roles in plant cell wall formation and lignification, and the production of ROS. | CAAGGTGAACTCGTGATGGA  TTGAGGATTCAACCGTCGTT | (Casassola *et al.*, 2015) |
| AF251217.1 –  *GAPDH* | Glyceraldehyde-3- phosphate dehydrogenase, housekeeping gene. | GGCCGGGATTGCTCTGAACG  TGGTGCTGTGCATGTGACGG | (Guo *et al.*, 2011) |
| *α-tubulin* | H α-tubulin, housekeeping gene. | ATCTCCAACTCCACCAGTGTCG  TCATCGCCCTCATCACCGTC | (Perochon *et al.*, 2015) |
