# Supplementary material for: A Microbial Fermentation Mixture Primes for Resistance Against Powdery Mildew in Wheat
Source: Front Plant Sci. 2019 Oct 9;10:1241. doi: 10.3389/fpls.2019.01241 (PMC6794463; doi:10.3389/fpls.2019.01241)
Supplement: Supplementary file 2 [file Image_1.pdf]

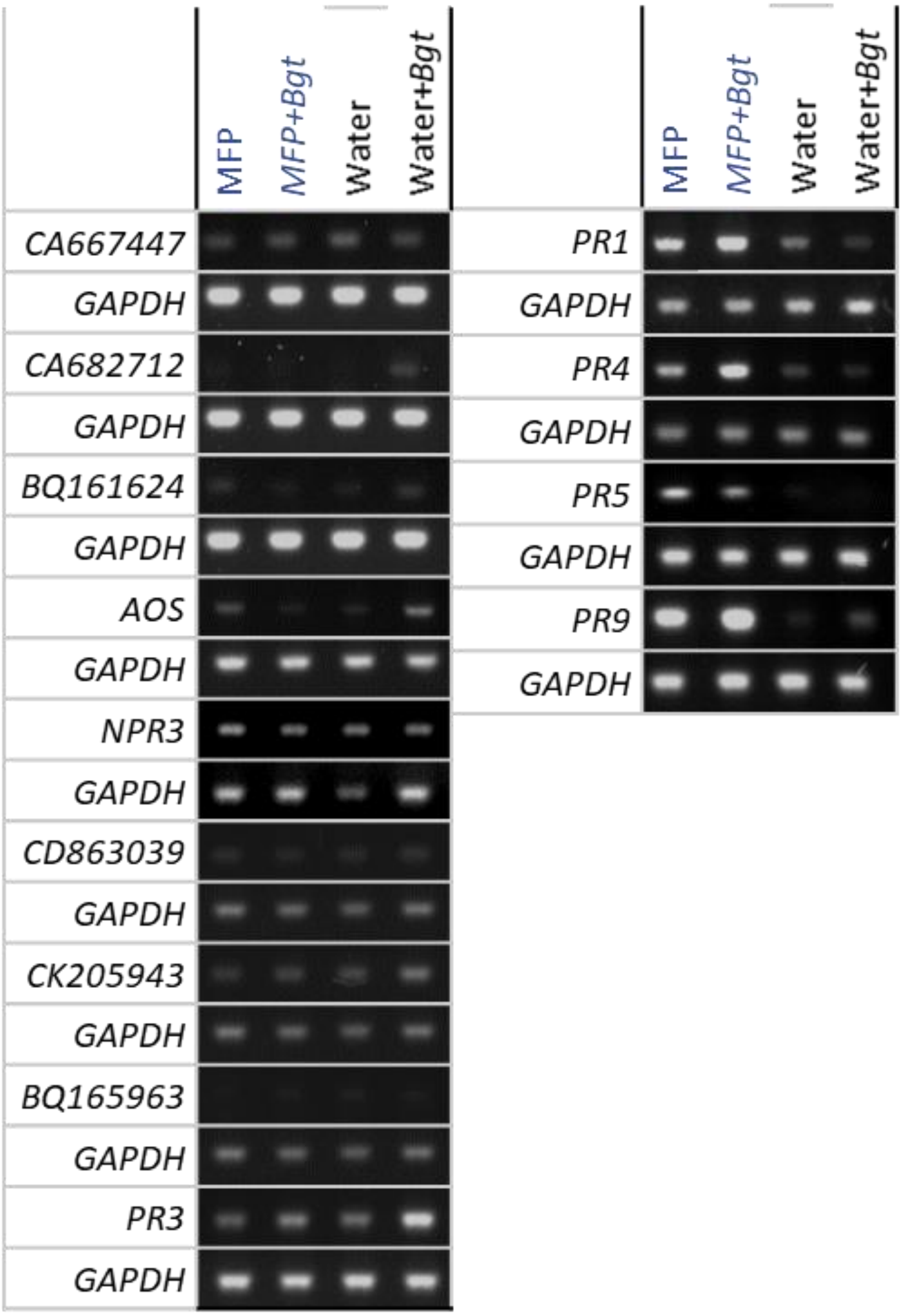

Supplementary Fig 1. RT-PCR analysis of defence related gene expression in wheat leaf tissue. Leaves of three-week old wheat cv. Avatar seedlings were treated with MFP and immediately inoculated with *B. graminis* f. sp. *tritici* (*Bgt*). Transcripts of *CA667447, CA682712*, *BQ161624*, *AOS*, *NPR3*, *CD863039*, *CK205943*, *BQ165963*, *PR3*, *PR1*, *PR4*, *PR5* and *PR9* compared to the housekeeping gene *GAPDH*. Tissue was harvested at 24 hpi (two leaves from two plants pooled as a biological sample) for analysis. Results shown are representative of two independent experiments.
